# Supplementary material for: Fatty Acid-Binding Proteins 4 and 5 Are Involved in the Pathogenesis of Retinal Vascular Diseases in Different Manners
Source: Life (Basel). 2022 Mar 23;12(4):467. doi: 10.3390/life12040467 (PMC9025502; doi:10.3390/life12040467)
Supplement: Supplementary file 1 [file life-12-00467-s001.zip › life-1628537-supplementary.pdf]

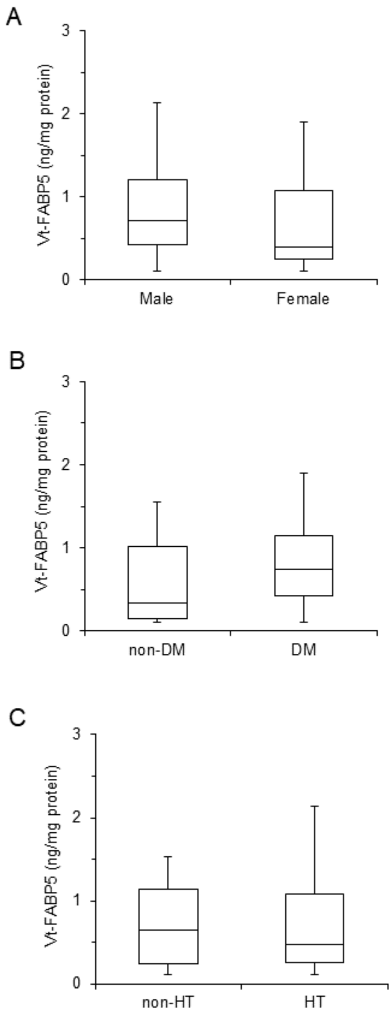

Figure S1. Vitreous concentrations of FABP 5 (Vt-FABP5), FABP4 (Vt-FABP4) and VEGFA (Vt-VEGFA) in patients with non-RVO or RVD.

Levels of Vt-FABP5 (ng/ml) were plotted between following groups; A) male (n=19) and female (n=29), B) non-DM (n=16) and DM (n=16), or C) non-HT (n=24) and HT (n=16), respectively.
